# Data on predictive resilience of interdependent water and transportation infrastructures: A sociotechnical approach

**DOI:** 10.1016/j.dib.2021.107512

**Published:** 2021-10-27

**Authors:** Babak Aslani, Shima Mohebbi

**Affiliations:** Department of Systems Engineering and Operations Research, George Mason University, Fairfax, VA USA

**Keywords:** Interdependent critical infrastructures, Resilience assessment, Natural disasters, Cascading random failures

## Abstract

Interdependent infrastructure systems are vulnerable to the cascading effect of failures resulting from random failures and natural disasters. The data provided in this work is the processed data used for the proposed resilience assessment framework for interdependent water and transportation networks dealing with both types of failure [1]. The case study is the interconnected networks of water and transportation in Tampa, Florida. The data for the random failure is obtained from the developed algorithmic framework and the land use and social vulnerability data provided by the U.S. Census datasets. We then used a subset of this produced data to construct predictive models for the network resilience to random failures. As for the natural disaster scenario, we focused on hurricane Irma in 2017 as it directly affected the focused region in Florida. We used the specific guidelines and the raw flooding data for this hurricane, provided by FEMA, to estimate the standing water for each geographical area (polygons) and the associated network components. We labeled the areas as failed and undamaged based on the estimated water levels. Finally, we used this data for developing a geospatial Geographical Weighted Regression (GWR) model to predict the resilience in each polygon. We present the final dataset for water and transportation networks to facilitate reusability for any future resilience study in the selected urban area.

**Specifications Table**

**Table:** 

Subject	Engineering
Specific subject area	Systems Engineering and Operations Research
Type of data	Table Figure
How data were acquired	The data related to land use and social vulnerability indicators were acquired from the U.S. Census dataset. Python was used as the primary programming language to process this data for the community detection step and generating the output of the developed resilience assessment framework. We also used Minitab software for the design of experiments conducted on the data. The detailed data for the consequent flooding of hurricane Irma in Florida was collected from the FEMA report. We used R to produce the resilience shapefiles for the given water and transportation networks.
Data format	Raw and Analyzed
Parameters for data collection	We limited the spatial data to the geographical boundaries of Tampa, Florida.
Description of data collection	We collected the water depth level data for Hurricane Irma in the state of Florida from FEMA and then partitioned the data to focus on Hillsborough County, which covers the whole region of Tampa. In the next step, we overlaid the water and transportation networks to the data and kept the areas inside these networks.
Data source location	City: Tampa, FL Country: USA Primary data sources: FEMA U.S. Census Data City of Tampa utilities Florida Department of Transportation (FDOT)
Data accessibility	https://doi.org/10.5281/zenodo.5565181
Related research article	Rahimi-Golkhandan, A., Aslani, B., & Mohebbi, S. (2021). Predictive resilience of interdependent water and transportation infrastructures: A sociotechnical approach. Socio-Economic Planning Sciences, 101166. https://doi.org/10.1016/j.seps.2021.101166

## Value of the Data

• The data set contains detailed information on consequent flooding of hurricane Irma in Tampa, Florida, as well as the estimated resilience of water-transportation networks based on a sociotechnical approach.
• The data set can be used by other researchers who work on resilience assessment of interdependent critical infrastructure systems.
• The data can be used for future research on comprehensive resilient assessment and social vulnerability in similar areas or the current region by adding other interconnected networks such as power and wastewater to the framework.
• This data set includes data for cascading failure triggered by both random failures, due to aging infrastructures, and natural disasters.

## 1 Data Description

The first file (DOE.csv) is the input data for the Taguchi Design of Experiment step. This data contains 24 columns and 70 observations (experiments). The first three columns represent the categorical variable of land use for the regions inside Tampa. The following three columns are the assigned community to each census block inside the interdependent network. The water and transportation columns show the magnitude (percentage) of failure in each network, respectively. The last column (resilience) is the output of the proposed algorithm for cascading failure in each experiment. Other columns also capture the interaction between the factors.

**Fig. 1:**
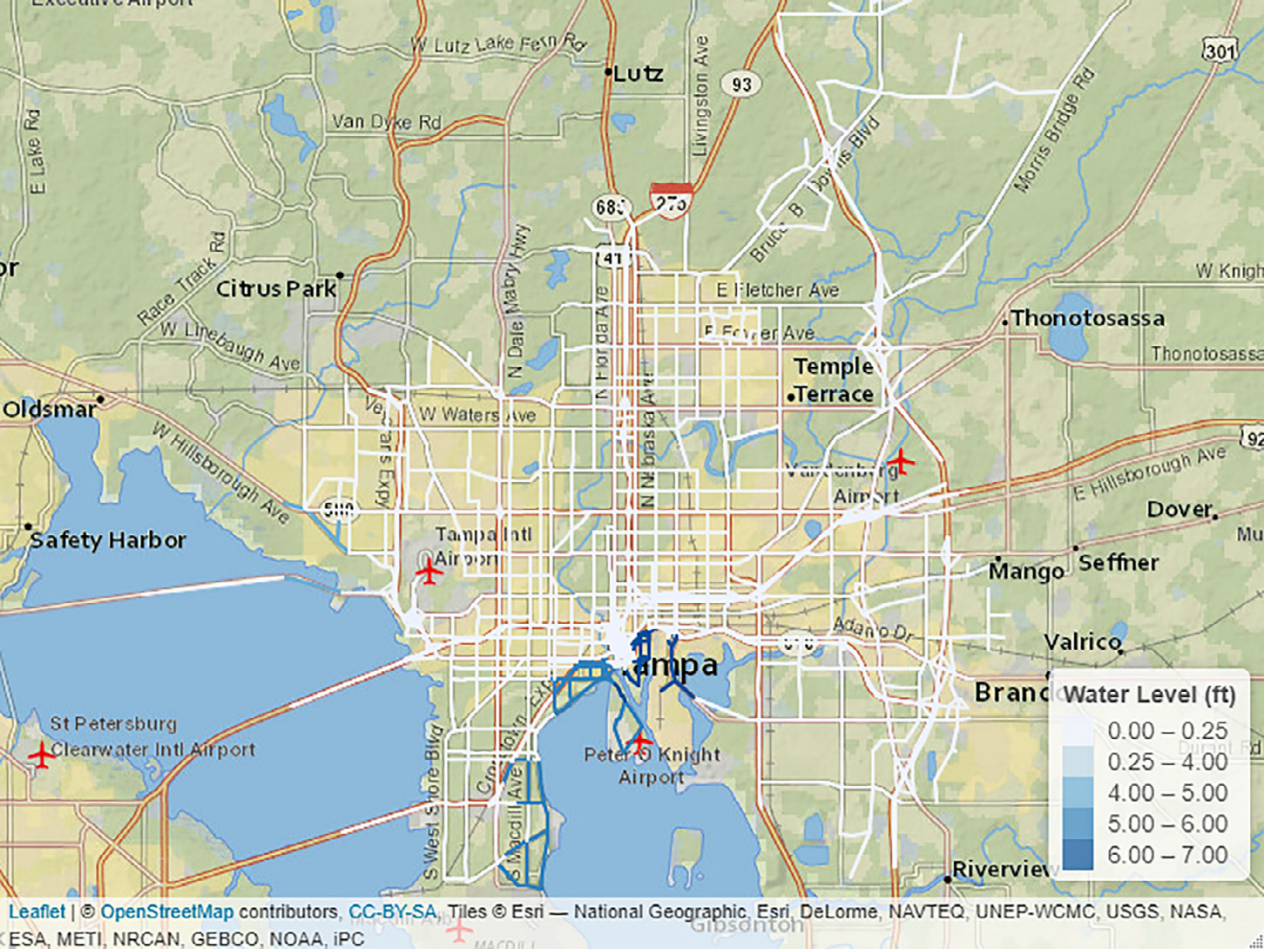
Standing water level for transportation network (in ft).

Fig. 1 visualizes the estimated water standing in each road section resulting after hurricane Irma. White road segments are untouched, and the affected intersections are colored with a spectrum of blue color proportionate to their water height.

**Fig. 2:**
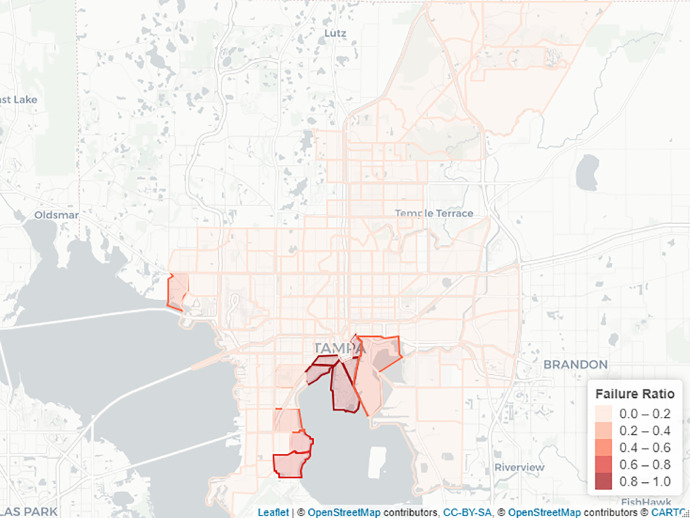
Failure ratio of water and transportation services.

Fig. 2 shows the failure ratio for each polygon in the City of Tampa as a result of hurricane Irma. These failures are the initial breakdowns within the interdependent networks. Failure ratio equal to 1 means that all the services provided by water and transportation networks are disrupted, while a zero value reflects that the area is unaffected by the flooding.

**Fig. 3:**
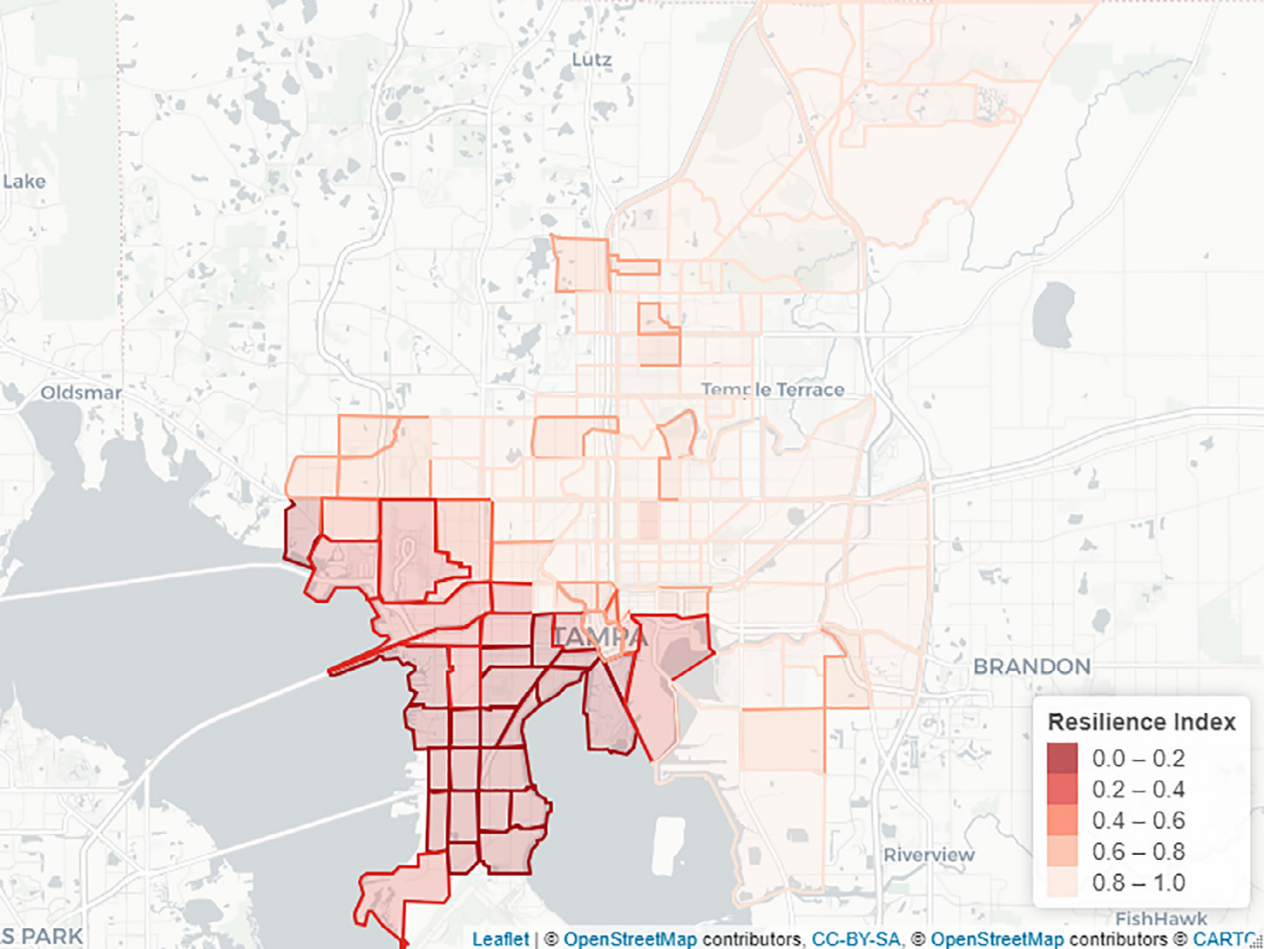
Resilience index map.

Fig. 3 shows the calculated resilience index in each polygon. The resilience index 1 means that the area remained intact to the cascaded failure resulting from initial breakdowns and both networks operate normally. However, a zero resilience index mirrors that the water and transportation services are entirely shut down in the region due to the cascading failure the flooding.

The second file (Water-Irma.csv) is the combination of the inputs provided by the City of Tampa utilities and the calculated standing water assigned to each pipe. We provide a brief description about each column for this data set of more than 1900 pipes in the water network.

• dc-id & node1& node2 : The ID for each pipe and the two connecting nodes.
• Waterlevel: Calculated standing water for each pipe.
• Initially-failed : The initially failed pipes.
• Cascaded-failed: The cascaded failed pipes.

The third file (Transportation-Irma.csv) contains the inputs from roadways and the calculated standing water assigned to each road segment. The information related to the transportation network is publicly available from an ArcGIS database developed by Florida Department of Transportation (FDOT)^1^. We provide a brief description about each column for this data set of more than 5400 intersections in the transportation network.

• OBJECTID: The ID for each road segment.
• CAPACITY & NUM-LANES: The capacity and number of lanes for each road segment, respectively.
• Waterlevel: Calculated standing water for each road.
• Initially-failed : The initially failed roads.
• Cascaded-failed: The cascaded failed roads.

The last file (Census blocks.csv) is the combination of the inputs collected from the U.S. Census Data and the proceed data from our analyses. The spatial information for polygons is based on the publicly available U.S. Census Data. A brief description about each column for this data set of 129 polygons is given below.

• GEOID /& NAMEL& dc-id & STATE & COUNT: The spatial information embedded in the shapefile of U.S. Census Data.
• ALAND & AWATE: Available land and water in each region, respectively.
• P0130 & H0100 & P0030 & P01202 & H0180 & H0040 & P0040 & H0020 & P008004 & P008005 & P01200 & P01204 & c-0-5-1 & c-0-5-3 & c-1-5-1-2 & c-1-5-1 & c-1-5-3-3 & c-1-5-3-4 & c-1-1-25-3 & c-1-1-25-4 & c-1-1-25-5 & c-1-2-125 & c-1-2-12 & c-1-3-3 & c-1-3-4 & c-1-3-5: The social vulnerability variables extracted from Social Vulnerability Index (SoVI) database.
• FaildRt: The failure ratio of water and transportation in each region.
• LandUse: The land use of each region.
• Community: The community of each region.
• Resilience: The calculated resilience for each region.

## 2 Experimental Design, Materials and Methods

We first used the SoVI data and conducted a community detection method to assign community profiles to water and transportation networks. Given the number of our factors and their levels, we designed a Taguchi DOE procedure in Minitab software to identify the significant factors and interactions for random disruption scenarios. Then, we used different combinations as the input to our developed algorithm (in Python) to simulate the cascading failure. We recorded the output of the algorithm, the resilience index, as the response variable. We used this data for the supervised predictive modeling of random failures in our study [1].

For the natural disaster, we focused on the flooding incident caused by hurricane Irma in 2017. We used the raw data for water depth levels in Florida to design our method. We used the nearest neighboring method in R to estimate the water levels for each polygon inside the boundaries of Tampa. To this end, we calculated the midpoints of the polygons in Tampa and the midpoints of the spatial polygons in the FEMA data set. Then, we calculated the Euclidean distance between these two sets of points and assigned each polygon to the closest water depth point. We implemented another nearest neighboring method to allocate each pipe and road to a polygon if their centers are located inside that region. In the next step, we assumed that all pipes and road segments inside each polygon have a similar standing water level. We appended the calculated water levels to the data related to water and transportation networks. We used the threshold suggested by FEMA, 0.25 ft, as the minimum water level to consider an area as flooded. We considered this failure as the initial failure resulted from flooding and used the cascading failure algorithm to track the propagation of failures in both infrastructures. Finally, we calculated the resilience index in each polygon based on the cascaded disruption. We defined the ratio of service preservation during the flooding as the resilience measure for both water and transportation. The resilience for both infrastructures was calculated, and the average value was marked as the overall resilience index.

## CRediT authorship contribution statement

**Babak Aslani:** Methodology, Software, Formal analysis, Data curation, Writing – original draft, Writing – review & editing, Visualization. **Shima Mohebbi:** Project administration, Funding acquisition, Conceptualization, Supervision, Methodology, Resources, Writing – review & editing, Validation.

## Declaration of Competing Interest

The authors declare that they have no known competing financial interests or personal relationships that could have appeared to influence the work reported in this paper.

## Footnotes

1 https://gis-fdot.opendata.arcgis.com/.
